# NRXN1 Deletion and Exposure to Methylmercury Increase Astrocyte Differentiation by Different Notch-Dependent Transcriptional Mechanisms

**DOI:** 10.3389/fgene.2019.00593

**Published:** 2019-06-21

**Authors:** Marilena Raciti, Jahan Salma, Stefan Spulber, Giulia Gaudenzi, Zahra Khalajzeyqami, Mirko Conti, Britt-Marie Anderlid, Anna Falk, Ola Hermanson, Sandra Ceccatelli

**Affiliations:** ^1^Department of Neuroscience, Karolinska Institutet, Stockholm, Sweden; ^2^Centre for Molecular Medicine, Department of Molecular Medicine and Surgery, Karolinska Institutet, Stockholm, Sweden; ^3^Department of Clinical Genetics, Karolinska University Hospital, Stockholm, Sweden

**Keywords:** neurotoxicity, methylmercury (MeHg), NRXN1 bi-allelic deletion, autism spectrum disorders, astroglia, notch signaling pathway

## Abstract

Controversial evidence points to a possible involvement of methylmercury (MeHg) in the etiopathogenesis of autism spectrum disorders (ASD). In the present study, we used human neuroepithelial stem cells from healthy donors and from an autistic patient bearing a bi-allelic deletion in the gene encoding for *NRXN1* to evaluate whether MeHg would induce cellular changes comparable to those seen in cells derived from the ASD patient. In healthy cells, a subcytotoxic concentration of MeHg enhanced astroglial differentiation similarly to what observed in the diseased cells (N1), as shown by the number of GFAP positive cells and immunofluorescence signal intensity. In both healthy MeHg-treated and N1 untreated cells, aberrations in Notch pathway activity seemed to play a critical role in promoting the differentiation toward glia. Accordingly, treatment with the established Notch inhibitor DAPT reversed the altered differentiation. Although our data are not conclusive since only one of the genes involved in ASD is considered, the results provide novel evidence suggesting that developmental exposure to MeHg, even at subcytotoxic concentrations, induces alterations in astroglial differentiation similar to those observed in ASD.

## Introduction

Epidemiological and experimental evidence points to a direct link between adverse events during early life and the pathogenesis of neurodevelopmental disorders (Grandjean et al., 1998; Rice and Barone, 2000; Seckl, 2004; Mueller and Bale, 2008; Onishchenko et al., 2008; Cottrell and Seckl, 2009; Perera and Herbstman, 2011; Tran and Miyake, 2017). Due to its peculiar physiological properties, the developing brain is particularly vulnerable to toxic insults (Saunders et al., 2000, 2012; Ek et al., 2012). As a result, *in utero* exposure to adverse prenatal environments induced by maternal stress, pharmaceutical compounds, or chemical pollutants, can disrupt normal neurodevelopment and lead to long-term detrimental effects on the nervous system structure and function (Lauwerys et al., 1978; Koger et al., 2005; Perera and Herbstman, 2011; Ceccatelli et al., 2013; Tran and Miyake, 2017; Raciti and Ceccatelli, 2018). Moreover, both experimental and clinical studies indicate that the developing brain is susceptible to toxicants even at exposure levels exerting no effects in adults (Palmer et al., 2006; Perera and Herbstman, 2011; Ceccatelli et al., 2013; Lanphear, 2015; Edoff et al., 2017).

Several chemicals, such as metals, pesticides, and endocrine disruptors, have been associated to neurodevelopmental disabilities including autism spectrum disorders (ASD), attention deficit hyperactivity disorder (ADHD) and cognitive impairments (Grandjean and Landrigan, 2014).

Among the environmental contaminants suggested to be possibly involved in the etiopathogenesis of ASD is methylmercury (MeHg) (Morris et al., 2018). MeHg represents a major concern because of its high neurotoxic potential on developing organisms (Ceccatelli et al., 2013). The developmental neurotoxicity of MeHg became evident after the Minamata catastrophe, occurred in Japan between 1950 and 1960, when symptom-free women gave birth to children with severe neurological disorders (Harada, 1995). Since then, a number of experimental studies reported that early developmental exposure to low concentrations of MeHg is associated with long-term behavioral deficits, cell death and impaired neurogenesis (Johansson et al., 2007; Onishchenko et al., 2007, 2008; Bose et al., 2012). In previous studies, we showed that prenatal exposure to MeHg in mice induces long-lasting impairments in learning capabilities, depression-like behavior and epigenetic changes at BDNF gene promoter, indicating epigenetic events as key mediators of MeHg effects (Onishchenko et al., 2007, 2008). Also epidemiological studies report a clear association between prenatal/early postnatal exposure to MeHg in fish-consuming populations and adverse neurodevelopmental outcomes, including neurological, cognitive and behavioral deficits (Harada, 1978; Grandjean et al., 1997, 1999; Murata et al., 1999, 2004; Jedrychowski et al., 2007; Grandjean and Landrigan, 2014; Strain et al., 2015).

The relationship between ASD and MeHg is still controversial and the available data is inconclusive (Morris et al., 2018). Many studies conducted in the last 30 years have found that mercury represents a risk factor for ASD (Palmer et al., 2006; Woods et al., 2010; Blanchard et al., 2011; Yoshimasu et al., 2014), and several of them showed a correlation between tissue mercury levels and ASD symptom severity (Holmes et al., 2003; Elsheshtawy et al., 2011; Lakshmi Priya and Geetha, 2011; Geier et al., 2012; Kern et al., 2016). However, there are also reports suggesting that MeHg should not be regarded as a risk factor for ASD (van Wijngaarden et al., 2013; Yau et al., 2014; McKean et al., 2015).

Both environmental and genetic factors are believed to play a role in the etiology of ASD, which is among the most complex neurodevelopmental disorders characterized by social and communication deficits, stereotyped behaviors and language impairments (Kim et al., 2008). Among the numerous genetic and chromosomal abnormalities that have been associated with autism pathogenesis, mutations in the gene encoding for neurexin 1 (*NRXN1*) have been identified as an ASD risk factor (Kim et al., 2008; Yan et al., 2008; Ching et al., 2010; Béna et al., 2013). Neurexins are a family of highly polymorphic cell surface proteins playing a crucial role in synapse formation, function and connectivity (Ching et al., 2010). In mammals, neurexins are encoded by three genes, *NRXN1*, *NRXN2* and *NRXN3*, each being transcribed from two independent promoters and generating two main isoforms, α and β (Béna et al., 2013). In humans, the role of NRXN1 in synaptic transmission during late neurogenesis has been extensively investigated (Zeng et al., 2013; Pak et al., 2015). Mutations in the *NRXN1* gene have been implicated in a variety of conditions including autism, schizophrenia, and nicotine dependence (Ching et al., 2010). More recently, a study by Béna et al. (2013) identified a bi-allelic deletion in *NRXN1* gene in a patient with ASD and provided evidence supporting a pathogenic role for heterozygous exonic deletions of *NRXN1* in neurodevelopmental disorders. The two inherited deletions found in the autistic donor are independent and partly overlapping, with a size of 0.18 and 0.40 Mb (Béna et al., 2013). Major clinical features found in the patient include ASD, intellectual disability, moderate motor developmental delay, language delay, seizures, hypotonia. Conversely, both parents (heterozygous carriers of the deletion) are phenotypically healthy (Béna et al., 2013).

By leveraging on the iPS cell technology (Takahashi et al., 2007), the Falk’s lab at Karolinska Institutet established a new cell model derived from the above mentioned autistic patient carrying a bi-allelic deletion in *NRXN1* gene (Béna et al., 2013), offering the possibility to address our questions on MeHg and ASD. We designed the present study to investigate whether subcytotoxic concentrations of MeHg (nanomolar range) would induce cellular/molecular alterations similar to those observed in cells originating from ASD patients. Using cells derived from healthy donors (H cells) and cells derived from the autistic patient (N1), we could show that in healthy cells MeHg increases the differentiation toward astroglia similarly to what occurs in the N1 diseased cells. Both in healthy and N1 cells, such alterations could be reversed by treatment with DAPT (*N*-[*N*-(3,5-difluorophenacetyl) -l-alanyl] -*S*-phenylglycine *t*-butyl ester), strongly suggesting that a misfunction of the Notch pathway may be a key common player underlying the defective differentiation, although via different transcriptional mechanisms.

## Materials and Methods

### Ethics Statement

Ethical permission for reprogramming human cells (Reprogrammering av mänskliga celler) dnr 2012/208-31/3 with addendum 2012/856-32 and 2015/1097-31/1 has been approved by the Ethical review board (Regionala etikprövningsnämnden i Stockholm). All methods were carried out in accordance with these approved guidelines and regulations. All samples were given with written informed consent. Samples from the autistic patient were given with written informed consent obtained from the parents.

#### Cell Culture Procedure

All experiments were carried out on neuroepithelial stem cell lines generated from human iPSC cells in the iPS core facility at Karolinska Institutet, as previously described (Falk et al., 2012). Briefly, skin fibroblasts obtained from healthy individuals (C3, C7) and one individual with ASD carrying bi-allelic *NRXN1* deletion were induced toward neuroepithelial stem (NES) cells and further differentiated spontaneously for 28 days. The experiments reported here were performed on C3 cells, whereas C7 cells were kept as backup. Plates coated with poly-L-Ornithine (0.1 mg/ml, Sigma) for 3 h at 37°C, were washed twice with PBS then coated with laminin (5 μg/ml, Sigma) at 4°C overnight. During the expansion step, healthy and autistic cells were cultured at the density of 45,000 cell/cm^2^ in proliferation media composed as follows: DMEM/F12 (1:1) + Glutamax, supplemented with Pen/Strept 1:100 (Life technology), N2 1:100 (Life technology), B27 1:1000 (Life technology), EGF (10 ng/ml, Invitrogen) and βFGF (10 ng/ml, R&D System). Half of the medium was replaced daily with fresh medium containing growth factors (GFs). Every 2–3 days, cells were passaged using TrypLE TM Express (1X Phenol red) and Defined Trypsin Inhibitor (1X) (Gibco). Cell suspension was collected in washing medium (DMEM/F12+ Glutamax), supplemented with 0.2% bovine serum albumin (BSA) and centrifuged 3 min at 1100 rpm.

To induce differentiation, dissociated cells were seeded at a density of 40.000 cells/cm^2^ on poly-L-Ornithine and laminin-coated 6 well-plates (VWR) or glass coverslips (placed in Nunclon^®^ Δ MultidisHES, 24 wells) in GFs-free differentiation medium, composed as follows: DMEM/F12 (1:1) supplemented with N2 (1:100), B27 (1:100), Pen/Strept (1:100).

From differentiation day (DD) 0 until DD7, half of the medium was replaced daily with fresh medium. From DD8 until DD20, medium was replaced every other day, and laminin 1:1000 was added to the medium from DD15. In the last differentiation week (DD21-DD28), half of the medium was replaced every 3 days.

#### Experimental Treatments

To investigate the effects of MeHg, proliferating cells (40.000 cell/cm^2^) were seeded in T75 flasks and, 24 h after seeding, exposed to 10 nM MeHg (CH3HgOH, ALFA, Johnson Matthey, Karlsruhe, Germany) for 48 h, in proliferation medium (Supplementary Figure S1a). After exposure, parental cells (P, directly exposed to MeHg) were passaged, and daughter cell generation 1 (G1, proliferating cells never directly exposed), were seeded in proliferation medium. After 24 h, cells were either transferred in differentiation medium, or kept in proliferation medium for 48 additional hours before being processed with different approaches depending on the experimental purpose (Supplementary Figure S1a). Preliminary experiments testing doses from 2.5 to 12.5 nM MeHg established 10 nM as a concentration not inducing apoptosis or ROS formation in our model (Supplementary Figures S1b,c). Apoptotic nuclei were detected by staining with Hoechst 33342, 1 μg/ml. For intracellular ROS level measurement, 80,000 G1 cells/well were plated in 96 multi-well plates, pre-coated as described above, and kept in proliferation state for 72 h before performing the assay. The intracellular ROS levels in MeHg-exposed and control cultures were determined by image-iT Live Green Reactive oxygen species detection kit (Invitrogen). Briefly, cells were washed with warm HBSS (1X) containing Ca^2+^ and Mg^2+^ and incubated for 25 min at 37°C with 25 μM carboxy-H2DCFDA. This molecule is able to permeate live cells and become fluorescent in presence of non-specific ROS. The non-fluorescent molecule is converted to a green-fluorescent form when the acetate groups are removed by intracellular esterases and oxidation (by the activity of ROS) occurs within the cell. Hoechst 1 μM was added to the carboxy-H2DCFDA staining solution during the last 5 min of incubation. The fluorescence intensity was determined using a Fluoroskan Ascent FL 2.6 fluoroscope (Thermo Scientific). The excitation and emission wavelengths were 485 and 538 nm for carboxy- H2DCFDA, and 355 and 460 nm for Hoechst. All experiments were performed in triplicates and repeated at least three times.

For DAPT treatment, differentiating cells were administered with 1 μM DAPT from DD21 (when GFAP mRNA start to be detectable by qRT-PCR) until DD28, when samples were harvested for RNA extraction or fixed for immunofluorescence. 0.0001% DMSO was used as a vehicle and the same concentration was also applied to control cultures and no signs of toxicity were observed. All experiments were performed in triplicates and repeated at least three times.

### Immunocytochemistry

After exposure, 40.000 cell/cm^2^cells were plated on glass coverslips placed in 24 well-plates (coated as already described). Cells were fixed in 4% paraformaldehyde for 25 min at room temperature and washed three times with PBS 1X for 5 min. After 1 min of permeabilization in Triton X-100 0.3%, cells were blocked with 10% goat serum (in PBS1X) at room temperature for 1 h and then incubated overnight at 4°C with primary antibodies diluted in 1.5% goat serum. The following day, samples were washed three times with PBS 1 X for 10 min at room temperature and subsequently incubated with the appropriate secondary antibodies AlexaFluor-488 or -594, for 2 h at room temperature. Primary and secondary antibodies and respective dilutions are reported in Table 1. Either DAPI or Hoechst 33342 have been used as nuclear counterstain; coverslips were mounted with Fluorescent Mounting Medium (Agilent Technologies, S302380-2). All fluorescence images were captured using a confocal microscope LSM 800 (Zeiss) or a fluorescent microscope (Nikon Eclipse Ti-S). For image analysis and quantification, images were collected from random fields (3–5 fields/well, *n* = 3 wells/condition in 2 biological replicates), and the programs ZEN 2.1 system, NIS Elements BR 3.2, or ImageJ/FiJi were used. FiJi (Version 2.0.0-rc-69/1.52n) was used to calculate the mean fluorescence. Briefly, for each image the total fluorescence of the different channels was calculated and normalized by the number of cells, detected by DAPI staining. The mean fluorescence value was averaged across experiments and normalized to control H cells. The number of cells expressing GFAP or TUJ1 was quantified using FiJi as follows: nuclei were identified in the blue channel (DAPI staining), using manually set threshold for each image individually. Mean intensities of GFAP and TUJ1 signal within the boundaries of nuclei were measured on the green and red channels, respectively. The signal intensity was scaled to range between 0 and 1, and the threshold for background noise in either channel was set to the 95th percentile of signal intensity in the other channel. The nuclei with signal intensity above the threshold were counted as GFAP or TUJ1 positive.

**Table 1:** Primary antibodies for immunofluorescence (IF) and Western blot (WB).

Primary antibody	Species	Dilution IF	Dilution WB	Company
NICD	Rabbit	1:100	1:1000	*Abcam, ab8925*
NESTIN	Mouse	1:200	1:1000	*Millipore, MAB5326*
HES5	Rabbit	1:50	–	*Nordic Biosite*
TUJ1	Rabbit	1:1000	–	*Nordic Biosite*, 802001/2
GFAP	Mouse	1:500	–	*Sigma, G*3893
α-tubulin	Rabbit	–	1:1000	Ab4074

### EdU Uptake Assay

Proliferating (G1) cells were grown on coverslips in a 24-well plate for 48h in presence of βFGF. Then cells were incubated with 10 μM 5-ethynyl-2′ì-deoxyuridine (EdU) for 90 min and fixed in 4% paraformaldehyde (PFA) (Sigma Aldrich) for 25 min at room temperature followed by washing with PBS. EdU visualization was performed using Click-iT EdU imaging kit (Invitrogen, Carlsbad, CA, United States) according to the manufacturer’s protocol. Fixed cells were incubated for 30 min with azide-conjugated Alexa Fluor 488 dye in Tris buffer supplemented with 4 mM CuSO4. Cells were then washed three times with PBS. For subsequent DNA staining, cell nuclei were counterstained with Hoechst 33342 for 5 min. After rinsing with PBS, coverslips were mounted onto slides with Vectashield mounting medium (Vector Laboratories, Inc., Burlingame, CA, United States). Images were collected from random fields by a Nikon inverted fluorescent microscope (Nikon Eclipse Ti-S) equipped with a Nikon Digital Sight DS-Qi1MC camera. For quantitative analysis, the images were batch processed (to avoid bias) using ImageJ.

### RNA Extraction, cDNA Synthesis and Quantitative RT-PCR

For gene expression analysis, proliferating and differentiating cells (40.000 cell/cm^2^) were seeded in 6 well-plate, coated as described above. Total RNA was extracted from at least 1 × 10^6^ cells using peqGOLD Total RNA kit (PEQLAB Biotechnologie GmbH, VWR) and the column DNase digestion (RNase-free DNase set; Qiagen VWR). Proliferating cells were harvested for RNA extraction 72 h after seeding; differentiating cells were harvested after 4 weeks of differentiation (Supplementary Figure S1a). RNA concentration and purification were determined by using a Nano Drop 1000 spectrophotometer (Thermo Scientific). cDNA was prepared using at least 1.5 μg of mRNA employing the Maxima first strand cDNA Synthesis for qRT-PCR (Thermo Fishers Scientific). Primers were designed for human target genes [see Table 2 for primer sequences and annealing temperature (Ta)]. Amplification reactions were performed in a final volume of 12.5 μl composed by 1 μl cDNA template (or 1 μl of water in the negative controls), SYBR Green PCR Master Mix and 0.2 μM of each primer. q-RT-PCR reactions were performed in QuantStudio 5 Real-Time PCR System, and the results were analyzed by Quant Studio^TM^ Design & Analysis Software 1.3.1 (Thermo Fisher Scientific).

**Table 2:** Primer sequences and the corresponding annealing temperatures (Ta).

Human target genes	Sequence FW	Sequence Rev	Ta (°C)
GFAP	GATCAACTCACCGCCAACAGC	CTCCTCCTCCAGCGACTCAATC	60
P16	GTGGACCTGGCTGAGGAG	CTTTCAATCGGGGATGTCTG	60
HES5	ACATCCTGGAGATGGCTGTC	AGCAGCTTCATCTGCGTGT	58
TUJ1	CTCAGGGGCCTTTGGACATC	CAGGCAGTCGCAGTTTTCAC	60
MAP2	GATGGAGTTCCACGATCAACAG	ACCAGGCTTACTTTGCTTCTCT	60
DCX	GCGAAATTTTTCAGGACCAC	CACAGAAGCCATCAAACTGG3	60
HPRT	ACCCCACGAAGTGTTGGATA	AAGCAGATGGCCACAGAACT	60

The amplification conditions were 20 s at 50°C, 10 min at 95°C, 15 s at 95°C and 1 min at the Ta for 40 cycles. To confirm the specificity of the qRT-PCR reactions, melting curve analyses were performed by adding a dissociation stage (conditions: 15 s at 95°C, 1 min at 60°C, 30 s at 95°C and 15 s at 60°C). The housekeeping gene hypoxanthine guanine phosphoribosyl transferase (HPRT) was used to normalize the expression levels of the target genes according to the following formulas:

ΔCT (difference threshold cycles) = CT of target gene – mean CT of housekeeping gene. To calculate the relative expression change we used the formula 2^−ΔΔCT^. All experiments were performed in triplicates and repeated at least three times.

### Western Blot

Protein extracts were prepared from at least 1 × 10^6^ proliferating cells in cell lysis buffer (Thermo-Scientific) supplemented with a protease inhibitor cocktail (Sigma-Aldrich). Proliferating cells were harvested 72 h after seeding, while differentiating cells were collected on DD28 (Supplementary Figure S1a). Protein concentrations were determined by BCA assay (Pierce) with BSA as standard. Equivalent amounts of total protein were diluted in sample buffer (sodium dodecyl sulfate-polyacrylamide) containing β-mercaptoethanol and boiled for 5 min. Twenty μg of protein were electrophoretically resolved on a 8–10% Tris-glycine gradient gels (Bio-Rad), then transferred to an Immobilon-P membrane (Millipore Inc.) using BioRad transfer system. After blocking with 5% skimmed milk in PBS for 1 h at room temperature, immunoblotting was carried out overnight at 4°C using appropriate primary antibodies: anti-NICD (1:1000, Abcam, 8925), anti-NESTIN (1:1000, Millipore, MAB5326) and anti-α-Tubulin (1:1000, Abcam, 4074), diluted with 5% milk in PBS. The following day, blots were incubated with the appropriate secondary horseradish peroxidase (HRP) anti-rabbit IgG (1:5000, Bio-Rad) for 2 h at room temperature followed by Chemiluminescence detection using Clarity Western ECL substrate Kit (Bio-Rad, 170-5061). The relative amount of protein was estimated using ImageStudio^TM^ Lite and normalized to beta-Actin. The experiments were repeated two times and performed in technical duplicates.

### Chromatin Immunoprecipitation

Chromatin immunoprecipitation (ChIP) was performed in triplicates, using iDeal ChIP-qPCR kit (Diagenode, Cat. No. C01010180) and according to the manufacturer’s instructions. Briefly, 10 μg of anti-NCOR antibody (Abcam 3482, Rabbit) and 10 μg of anti-H3K4me3 (Cell Signaling, Rabbit) were used respectively in each IP. Purified, sheared (Bioruptur, Diagenode) eluted DNA and 1% DNA Input were analyzed by room temperature-qPCR using SYBR Green PCR Master Mix (Life Technologies, K0222), following the instruction manual. Site-specific primers for human GFAP promoter [Qiagen, Cat. No., GPH1019679(-)02A], human HES1 promoter [Qiagen, Cat. No., GPH1009779(-)02A], and human HES5 promoter [Qiagen, Cat. No., GPH1014204(-)02A] were used to detect the enrichment. All experiments were performed in triplicates and repeated at least three times.

### Statistical Analyses

All gene expression data are presented as mean of 2^−ΔΔCT^ ± standard error of the mean. Non-parametrical statistical tests were used for data analysis (Kruskall–Wallis test for multiple comparisons followed by Mann–Whitney *U*-test for between-group comparisons). The significance value was set at *p* < 0.05.

## Results

### MeHg Increases Differentiation Toward Glia, as Occurring in N1 Cells

**FIGURE 1:**
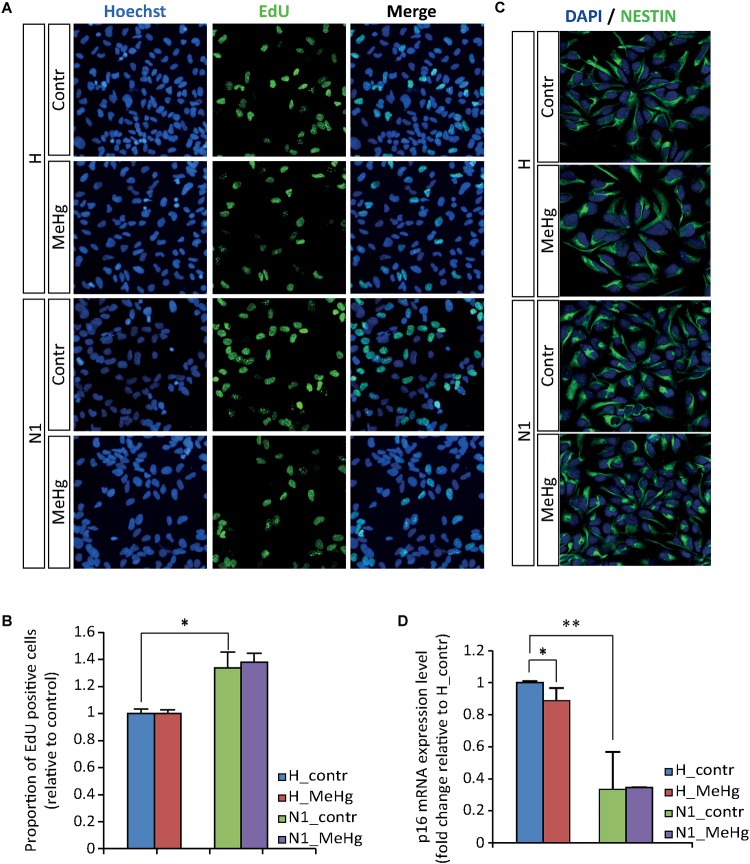
MeHg does not alter the proliferation rate. **(A,B)** EdU uptake assay performed in proliferating healthy (H) and N1 cells after exposure to 10 nM MeHg. The proportion of EdU-positive cells are normalized on untreated H (*n* = 3 experiments). **(C)** Illustration of ubiquitous expression of the stem cell marker NESTIN in proliferating H and N1 cells. **(D)** *P16* gene expression analysis evaluated by qRT-PCR in proliferating cells after MeHg exposure (*n* = 4 experiments). ^∗^*p* < 0.05 and ^∗∗^*p* < 0.01, Mann–Whitney *U* test.

In the attempt to evaluate whether MeHg exposure could induce alterations in healthy cells similar to those observed in cells derived from ASD patient (N1), we started by investigating cell proliferation and differentiation. We did not observe any significant difference in the percentage of EdU-positive cells between the untreated and MeHg-exposed cells in either group (healthy and N1 cells) (Figure 1A,B). In accordance, the immunoreactivity of the stem cell marker NESTIN appeared not to be affected by MeHg in both healthy and N1 cells (Figure 1C). Interestingly, there was a significantly higher proliferation rate in untreated N1 cells as compared to untreated healthy cells (Figure 1B). Hence, qRT-PCR analysis revealed that cyclin-dependent kinase inhibitor p16 is downregulated in N1 cells (Figure 1D).

**FIGURE 2:**
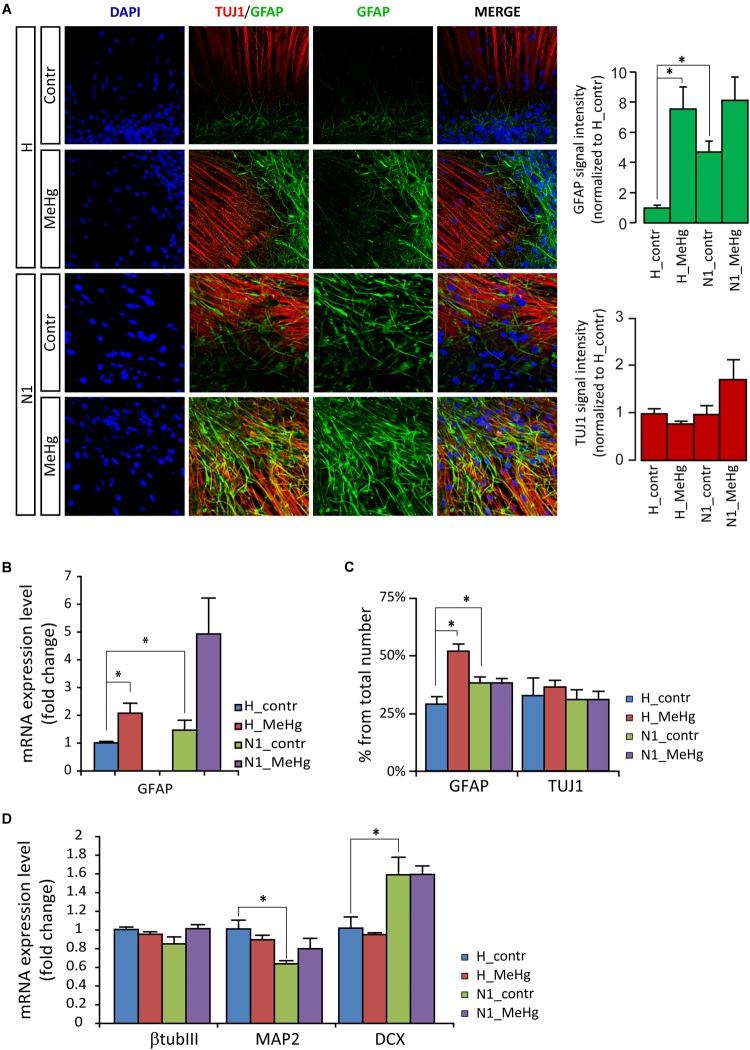
MeHg increases astroglial cell differentiation in DD28 cells. **(A)** DD28 cells immunostaining for DAPI (blue), the neuronal progenitor marker III β-tubulin (TUJ1, red), and the astrocytic marker glial fibrillary acidic protein (GFAP, green). The expression of GFAP is higher in N1 as compared to healthy (H) cells and is increased after MeHg exposure in DD28 cells, while the expression of TUJ1 is not affected (signal intensity quantification shown in the panels to the right) (*n* = 2 experiments). **(B)** The mRNA expression for GFAP is also higher in N1 than in H cells and is increased after MeHg exposure. ^∗^*p* < 0.05, Mann–Whitney *U* test. **(C)** The number of GFAP-positive cells is higher in control N1 than in control H cell and is increased by MeHg exposure only in H cells, while the number of TUJ1-positive cells is constant across groups. ^∗^*p* < 0.05, Mann–Whitney *U* test. **(D)** Neuronal markers expression assessed by qRT-PCR. MeHg exposure has no significant effects on the expression of *TUJ1*, *MAP2* and *DCX* are observed in healthy cells or N1 cells. The expression of *MAP2* is decreased, whereas *DCX* expression is increased in untreated N1 cells as compared to untreated healthy cells (*n* = 4 experiments/group). ^∗^*p* < 0.05, Mann–Whitney *U* test.

Gene expression and protein analyses in 4 weeks differentiated cells (DD28) showed that in healthy cells MeHg induced a strong increase of *GFAP* and GFAP positive cells, similarly to what was observed in N1 cells (not exposed to MeHg) (Figure 2A–C). Conversely, in both healthy and N1 cells, MeHg did not induce any significant change in the earliest phase neuronal differentiation marker β-TubulinIII (as detected by the antibody TUJ1 and qRT-PCR) (Figure 2A,C,D). Also, the post mitotic neuron marker Microtubule-associated protein 2 (*MAP2*) and the marker for neuronal precursor cells and immature neurons Doublecortin (*DCX*) mRNA levels were not affected by MeHg (Figure 2D). Interestingly, N1 cells exhibited a reduced expression of *MAP2* mRNA and increased *DCX*, suggesting that there might be a higher percentage of newborn neurons and a lower number of post mitotic neurons in N1 cultures (Figure 2D).

### MeHg Exposure Alters Notch1 Signaling Activation in DD28 Cells

**FIGURE 3:**
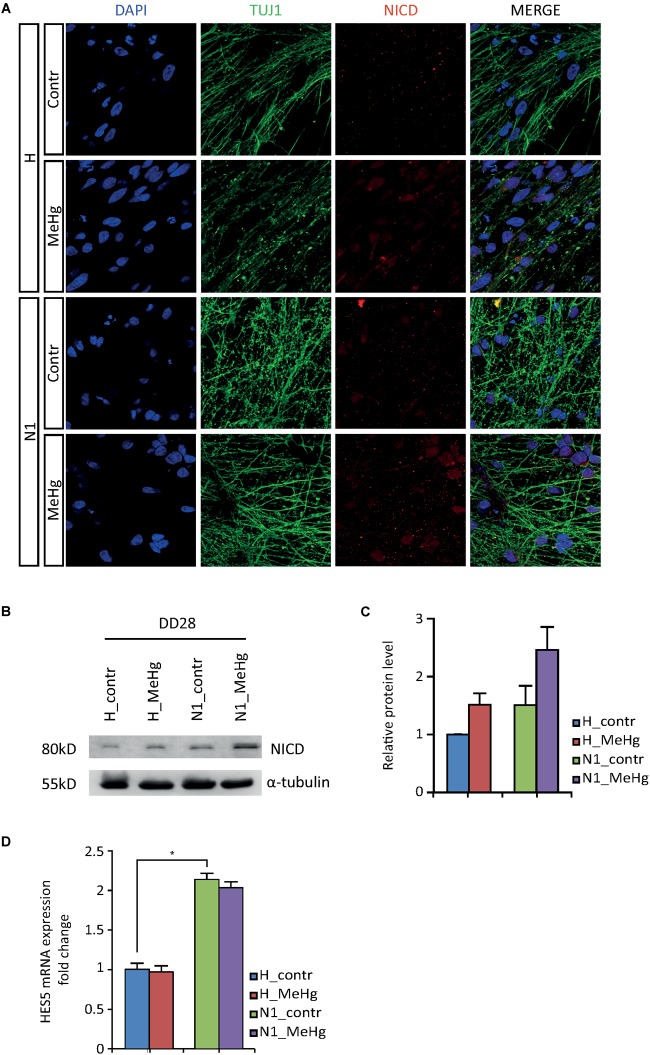
NICD and HES5 levels in DD28 healthy and N1 cells. **(A)** The NICD immunofluorescence signal is higher in DD28 healthy (H) exposed to MeHg and N1 cells; MeHg further increases NICD in N1 cells. **(B)** The Western blot analysis for NICD supports the immunofluorescence data as shown by the stronger 80 kD bands in H-MeHg cells, N1 cells, and N1-MeHg cells. All gels were run in the same experimental conditions (full-length blots of each tested protein are reported in Supplementary Figure S2; *n* = 2 experiments). **(C)** Quantification of relative protein levels for NICD. **(D)** *HES5* mRNA expression level in DD28 cells is higher in N1 cells as compared to H cells and is not is not affected by MeHg. ^∗^*p* < 0.05, Mann–Whitney U test.

In an attempt to elucidate the molecular mechanisms mediating the observed overproduction of astroglial cells, we first looked at Notch intracellular domain (NICD) at DD28. Immunoblotting data showed an increase in NICD after MeHg exposure, which was confirmed by immunofluorescence (Figure 3A–C). As shown in Figure 3A–C, the NICD level in MeHg-exposed healthy cells was comparable to the level observed in untreated N1 cells at DD28, suggesting that aberrant Notch signaling may be a common denominator mediating the glia overproduction observed in both MeHg-treated healthy cells and N1 untreated cells. Markedly, the expression of the Notch1 effector enhancer of split 5 (*HES5*) appeared to be high in N1 and was not affected by MeHg in either healthy, or N1 DD28 cells (Figure 3D).

### DAPT Counteracts MeHg Effect on Glial Differentiation in DD28 Healthy and N1 Cells

**FIGURE 4:**
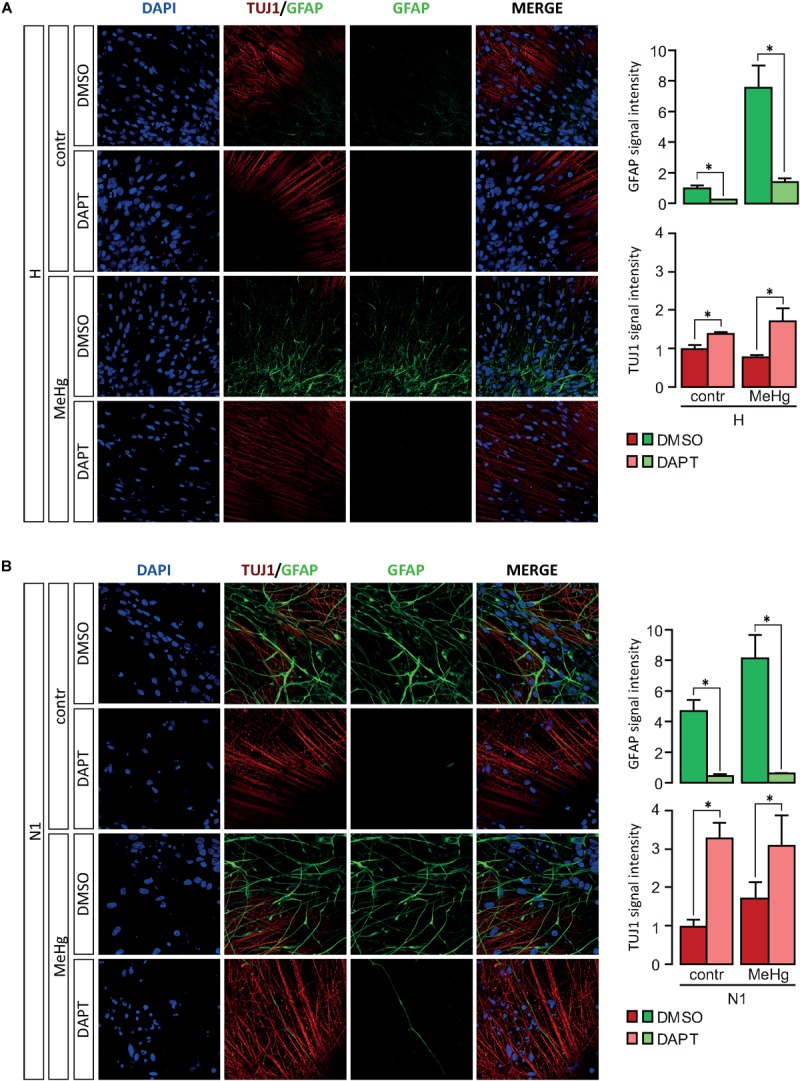
Expression of GFAP and TUJ1 following DAPT treatment in DD28 cells. **(A)** DAPT treatment prevents the MeHg-induced increase in GFAP immunoreactive H cells (*n* = 2). **(B)** In N1 cells, DAPT administration reduces the expression of GFAP in both untreated, and MeHg-exposed N1 cells (*n* = 2). Relative signal immunofluorescence signal intensity quantification is show in the panels on the right. ^∗^*p* < 0.05, Mann–Whitney *U* test.

Our findings hitherto suggested a role for Notch signaling in the alterations of differentiation observed in both MeHg-exposed healthy cells and N1 cells. To test this hypothesis, we pursued a combination treatment with Notch blockade in differentiating healthy and N1 cells, in the presence/absence of MeHg treatment. As *GFAP* gene starts to be expressed at high level after 3 weeks of undirected differentiation (data not shown), DD21 cells were administered with 1 μM DAPT and then immunostained for GFAP at DD28. Notch inhibition in DD21 cells counteracted the MeHg-induced astrocytes production in healthy cells as well as N1 cells (Figure 4A,B) and resulted in a dramatic decrease of GFAP immunoreactivity in both MeHg-exposed healthy and N1 cells (Figure 4A,B). Also TUJ1 immunoreactivity was affected by DAPT, as shown by the increase in staining intensity in both healthy and N1 cells (Figure 4A,B).

### MeHg Decreases N-CoR Occupancy at GFAP Promoter in Healthy, but Not in N1 Cells

**FIGURE 5:**
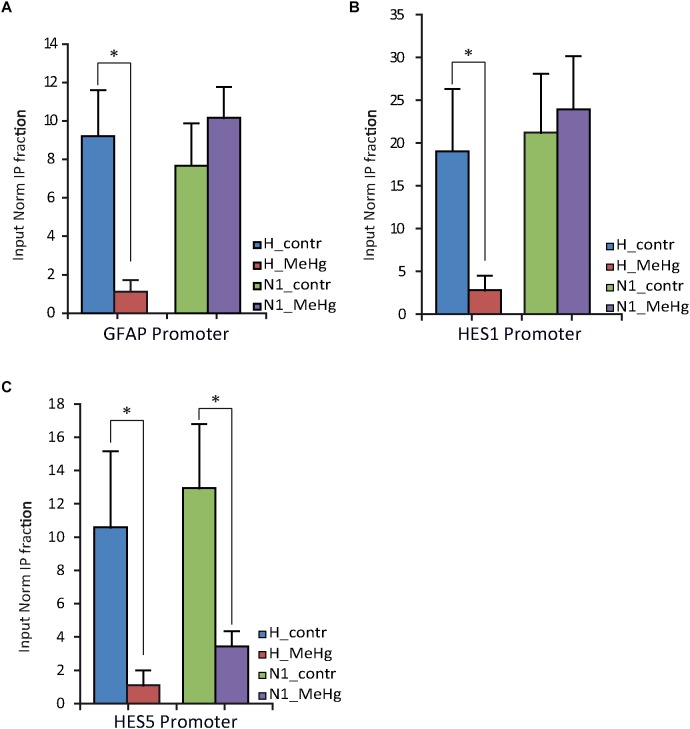
NCOR occupancy of *GFAP*, *HES1* and *HES5* promoters of H and N1 cells assessed by ChIP-qPCR after 21 days in differentiation conditions (DD21). **(A,B)** MeHg exposure significantly reduces the occupancy of N-CoR at *GFAP* and *HES1* promoter in H, but not in N1 cells. ^∗^*p* < 0.05, Mann–Whitney *U* test. **(C)** Significant decrease in N-CoR occupancy at *HES5* promoter site was observed in both control (H) and N1 cells exposed to MeHg. ^∗^*p* < 0.05, Mann–Whitney *U* test (*n* = 3 experiments).

The nuclear receptor co-repressor (N-CoR) binds *in vivo* to the Notch effector RBP-J; this complex, in turn, binds to the *GFAP* promoter leading to its transcriptional repression (Hermanson et al., 2002; Sardi et al., 2006; Andreu-Agulló et al., 2009). We therefore investigated N-CoR occupancy at *GFAP* and *HESs* genes promoters in healthy and N1 cells after 3 weeks of undirected differentiation (DD21). Interestingly, we found the N-CoR occupancy at the promoters of *GFAP* and the CSL/RBP-J target *HES1* to be strongly decreased in healthy cells treated with MeHg (Figure 5A,B) (GFAP, *p =* 0.030), suggesting that MeHg exposure directly activates the expression of *GFAP/HES1* through the Notch signaling activation and the following N-CoR-mediated transcriptional de-repression. On the other hand, we could not find any significant difference in N-CoR occupancy at *GFAP/HES1* promoters in N1 cells or N1 MeHg-exposed cells (Figure 5A,B), suggesting that there is a different transcriptional mechanism underlying the astrocytes overproduction observed in these cells.

### HES5 Expression in Proliferating Cells

**FIGURE 6:**
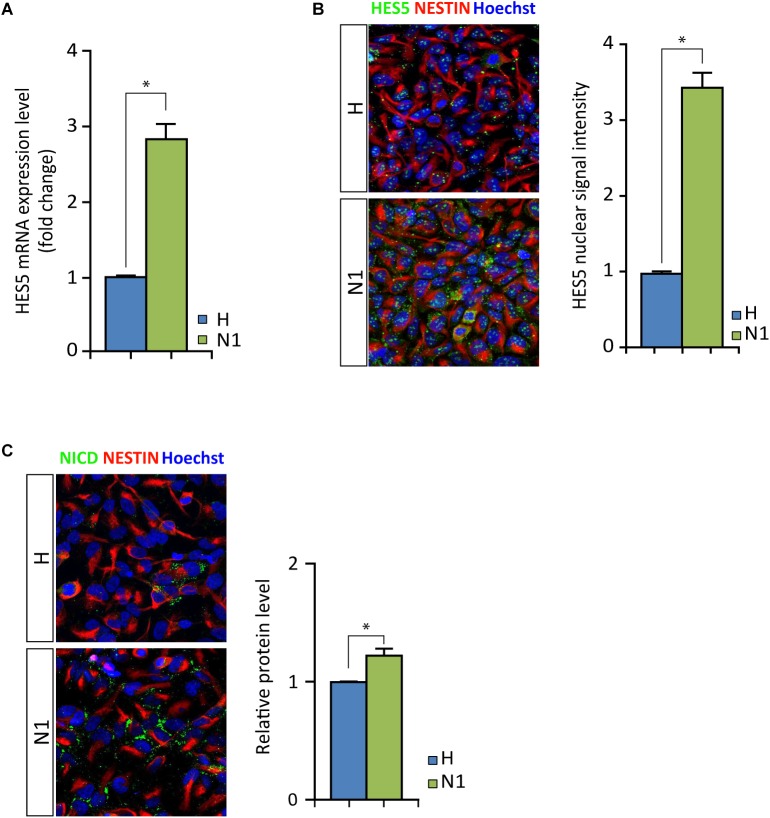
HES5 and NICD basal levels in untreated proliferating healthy and N1 cells. **(A)** *HES5* mRNA expression level is higher in N1 cells as compared to H cells. ^∗^*p* < 0.05, Mann–Whitney *U* test. **(B)** Fluorescence measurement of HES5 immunostaining in nuclei show an increased signal in proliferating untreated N1 cells. ^∗^*p* < 0.05, Mann–Whitney *U* test. **(C)** NICD (green) immunostaining in healthy (H) and N1 cells show an increase in NICD production in N1 cells, also confirmed by Western blot measurement (*n* = 2 experiments). *p* < 0.05, Mann–Whitney *U* test.

As mentioned above, we noted increased levels of *HES5* expression in N1 cells compared to healthy cells, irrespective of MeHg treatment (Figure 3D). We assessed the N-CoR occupancy at *HES5* promoter and found it to be decreased by MeHg treatment in both healthy and N1 cells (Figure 5C). Thus the N-CoR occupancy was dissociated from the increase in astroglial differentiation induced by MeHg. We therefore speculated that the increased *HES5* expression in the N1 cells could be linked to the increased gliogenesis, as a result of an imbalance in the early commitment of neural progenitor cells, and investigated *HES5* gene expression and nuclear levels in untreated cells. Both gene expression analysis and immunofluorescence of the nuclear levels showed significantly higher HES5 expression in N1 cells as compared to the healthy control cells (Figure 6A,B). Moreover, immunostaining and Western blot experiments in untreated cells demonstrated a higher basal level of NICD in untreated N1 as compared to untreated healthy cells (Figure 6C).

## Discussion

Although the role of genetic defects in ASD is well established, recent data point to a potential contribution by adverse prenatal events, such as exposure to environmental contaminants (Leslie and Koger, 2011; Karimi et al., 2017). A major concern in this context is represented by MeHg, a widespread environmental toxicant known to be highly toxic for the developing nervous system even at low concentrations that do not affect the adult brain (Harada, 1978, 1995; Kjellstroöm et al., 1989; Grandjean et al., 1997; National Research Council (US) Committee on the Toxicological Effects of Methylmercury, 2000; Myers et al., 2003; Albores-Garcia et al., 2016). Despite major actions taken to reduce its use and emission in the environment, MeHg contamination is still a persistent problem: after entering the aquatic food chain, MeHg accumulates mostly in large fish and sea mammals, whose consumption represents the main route of exposure in humans (Morel et al., 1998).

Even though MeHg is among the environmental chemicals suggested to be a risk factor for ASD, the available data are controversial (Kern et al., 2016; Ye et al., 2017; Morris et al., 2018). In the present study, we used human neuroepithelial stem cells derived from healthy donors and from an autistic patient carrying a bi-allelic deletion in *NRXN1* gene, to evaluate whether low concentrations of MeHg relevant for human exposure, induce alterations in neural differentiation comparable to those observed in cells derived from ASD patients (N1 cells).

Cells were exposed to 10 nM MeHg, a concentration derived from preliminary tests of doses from 2.5 to 12.5 nM MeHg, which does not induce apoptosis. The tested doses came from our previous experiments performed in human progenitor cells originating from fetuses of 8.5 and 16 postconceptional weeks where concentrations ranging from 2.5 to 100 nM were tested. Also in these two human cell models, 10 nM MeHg did not induce cytotoxicity (Edoff et al., 2017). Considering that the MeHg mean concentrations found in maternal blood in various countries range from 0.46 to 4.05 microg/L (Björnberg et al., 2003; Schober et al., 2003; Bjornberg et al., 2005; Donohue et al., 2018), the concentration used in our study (2.15 microg/L MeHg) is relevant for human exposure.

Exposure to 10 nM MeHg, induced alterations in undirected differentiation of healthy neuroepithelial stem cells, leading to an increased production of astrocytes, but no significant effects on the neuronal compartment. Other cell populations not included in our study may be also affected by MeHg. These results are in accordance with previous studies showing that both rodent and human neural stem cells (NSCs) are highly susceptible to MeHg and that even nanomolar concentrations can induce long-lasting impairments (Tamm et al., 2006, 2008; Moors et al., 2009; Theunissen et al., 2010; Bose et al., 2012). In this study we could not find any change in the proliferation capability in either healthy, or diseased N1 cells after MeHg exposure, as shown by the EdU uptake assay. This is consistent with our previous data showing that exposure to 10 nM MeHg does not affect proliferation of post conception week (PCW) 8.5-derived human neuronal progenitor cells (NPCs) (Edoff et al., 2017). As mentioned above, 10 nM MeHg shifted the differentiation toward an astroglial fate in healthy cells, and similar alterations were found in cells derived from the ASD patient (N1 cells), as shown by significantly higher levels of the astrocytic intermediate filament gene/protein GFAP. This is particularly relevant as according to previous studies brains from patients with ASD display higher glial marker expression (Laurence and Fatemi, 2005; Edmonson et al., 2014; Gandal et al., 2018). Specifically, GFAP levels have been shown to be increased in the cerebellum, cortex and cerebrospinal fluid of ASD patients (Ahlsén et al., 1993; Purcell et al., 2001; Laurence and Fatemi, 2005; Edmonson et al., 2014). The role of glial cells in ASD pathophysiology is further supported by transcriptomic analysis revealing an enrichment in astrocytes modules in ASD brains (Voineagu et al., 2011; Gandal et al., 2018).

In the developing mammalian neocortex, gliogenesis occurs after neurogenesis and it is finely regulated by the activity of several pro-gliogenic extracellular signals, including BMP, CNTF and Notch signaling (Zhou et al., 2010). The Notch signaling pathway is a major determinant of NSC cell fate, well-known to be critically involved in the regulation of NSC neurogenic to gliogenic “switch” (Morrison et al., 2000; Grandbarbe et al., 2003). Notch activation results in the release of NICD, which, after interacting with the DNA binding protein CSL, translocates into the nucleus. Here, the so-formed complex triggers the expression of *HESs* genes and *GFAP* (Zhou et al., 2010). *HES1* and *HES5*, the main Notch effectors, can promote gliogenesis by hampering the function of proneural genes (*NGN1, NGN2, MASH1*) or by cross-talking with JAK-STAT pathway (Zhou et al., 2010). Previous studies identified Notch as a potential target for MeHg toxicity (Bland and Rand, 2006; Tamm et al., 2008; Edoff et al., 2017). In rat and drosophila, MeHg can activate the Notch signaling pathway through regulation of ADAM metalloproteases (Bland and Rand, 2006; Tamm et al., 2008). Both in rat NSCs and human PCW 8.5 NPCs, MeHg-induced Notch activation stalls neuronal differentiation and forces neural progenitors in an undifferentiated state (Tamm et al., 2008; Edoff et al., 2017). We therefore investigated Notch activity in our cultures and detected a clear Notch overactivation in DD28 healthy cells exposed to MeHg as well as in N1 cells. MeHg further increased the NICD signal in N1 cells, strongly suggesting that a Notch misfunction could underlie the observed astrocytes overproduction in both healthy and diseased cells. Moreover, it could explain the higher proliferation capability observed in untreated N1 cells. In support of this hypothesis, both gene expression analysis and immunofluorescence data showed much higher HES5 levels in N1 as compared to healthy cells. In N1 cells additional mechanisms are likely to contribute to the increased proliferation rate, such as the dramatic downregulation of *p16*, which is known to block the cell cycle progression from G1 phase to S phase (Rayess et al., 2012).

Next, to investigate the link between Notch misactivation and increased astrocytes output, we looked at GFAP expression level and protein upon DAPT-mediated Notch inhibition. DAPT efficiently blocks the presenilin–γ-secretase complex (Dovey et al., 2001) and, consequently, prevents the Notch response activation (Geling et al., 2002; Crawford and Roelink, 2007). DAPT treatment restored a normal GFAP level further supporting the idea that misfunction of the Notch pathway plays a critical role in the altered differentiation observed in untreated N1 cells and in healthy cells exposed to MeHg cells. As expected the Notch inhibition by DAPT promoted neuronal differentiation as shown by the increased TUJ1 levels in both healthy and N1 cells.

It is well-established that N-CoR binds *in vivo* to the Notch effector CSL/RBP-J and the so-formed complex, in turn, directly mediates the transcriptional repression of the GFAP promoter (Hermanson et al., 2002; Sardi et al., 2006; Andreu-Agulló et al., 2009). Such N-CoR-induced repression can be relieved by NICD binding following Notch activation (Giaimo et al., 2017). Accordingly, we found a strong decrease of N-CoR occupancy at the promoters of *GFAP* and *HES1* in healthy cells exposed to MeHg, suggesting that the dramatic increase in *GFAP* expression could be directly mediated by a de-repression of the *GFAP* gene downstream to the MeHg-induced Notch activation. In contrast, N1 cells did not show any significant difference in N-CoR occupancy at *GFAP* or *HES1* promoters as compared to healthy cells, suggesting that different transcriptional mechanisms are involved in the abnormal astroglial differentiation observed in diseased cells. We reasoned that the higher HES5 expression previously noticed in the N1 cells could imbalance the early commitment of neural progenitor cells, resulting in increased gliogenesis. Consistently, gene expression and immunofluorescence experiments conducted in untreated proliferating cells clearly showed HES5 levels to be much higher in N1 cells as compared to healthy cells, suggesting that the Notch signaling could be overactivated. In support of this, we found higher basal level of NICD in untreated N1 cells, further pointing to an aberrant Notch activity in these cells.

## Conclusion

Our data show that the alterations in astroglial differentiation found in healthy cells after exposure to a subcytotoxic dose of MeHg are similar to those observed in cells derived from an autistic patient (N1). In both conditions, there is an increase in GFAP-positive cells and aberrant increase in the activity of the Notch signaling pathway. However, the similar phenotypes observed in MeHg-treated healthy cells and N1 cells appear to be associated with different transcriptional mechanisms. Although our conclusions should be strengthened by further analyses in cells derived from other type of ASD patients, the results provide novel evidence suggesting that developmental exposure to MeHg, even at low concentrations, relevant for human exposure, induces alterations in astroglial differentiation similar to the ones observed in cells derived from ASD patients carrying NRXN1 deletion.

## Author Contributions

MR, OH, AF, and SC contributed to the conception and study design. B-MA provided patient material. MR and JS involved in the cell culture experiments and contributed to the immunofluorescence and microscopy. MR and ZK contributed to the gene expression analysis. GG, MC, MR, and JS contributed to the ChIP. SS and GG contributed to quantification. JS and MR contributed to the Western blot. MR drafted the manuscript. MR, SC, SS, and GG edited and revised the manuscript.

## Conflict of Interest Statement

The authors declare that the research was conducted in the absence of any commercial or financial relationships that could be construed as a potential conflict of interest.
