# Supplementary material for: NRXN1 Deletion and Exposure to Methylmercury Increase Astrocyte Differentiation by Different Notch-Dependent Transcriptional Mechanisms
Source: Front Genet. 2019 Jun 21;10:593. doi: 10.3389/fgene.2019.00593 (PMC6610538; doi:10.3389/fgene.2019.00593)
Supplement: Supplementary file 1 [file Data_Sheet_1.pdf]

NRXN1 deletion and exposure to methylmercury increase astrocyte differentiation by different Notch-dependent transcriptional mechanisms

Marilena Raciti[1,*], Jahan Salma[1], Stefan Spulber[1], Giulia Gaudenzi[1], Zahra Khalajzeyqami[1], Mirko Conti[1], Britt-Marie Anderlid[2, 3], Anna Falk[1], Ola Hermanson[1], Sandra Ceccatelli[1, *]

[1] Department of Neuroscience, Karolinska Institutet, 171 77 Stockholm, Sweden
[2] Department of Molecular Medicine and Surgery, Centre for Molecular Medicine, Karolinska Institutet
[3] Department of Clinical Genetics, Karolinska University Hospital, Stockholm, Sweden.

* Corresponding Authors
Sandra.Ceccatelli@ki.se
marilenaraciti1@gmail.com

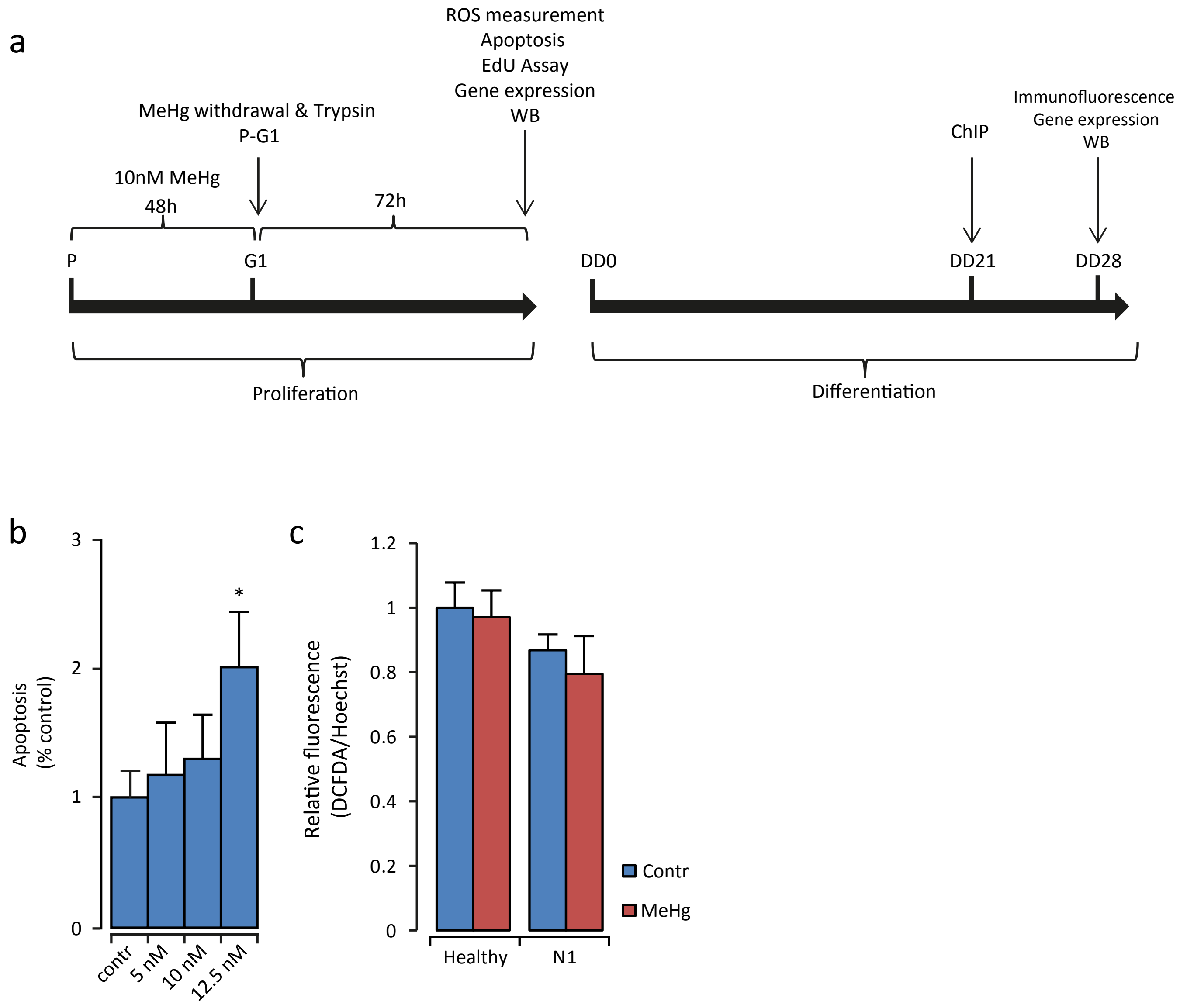

Supplementary Figure S1. a) Experimental design. Healthy and N1 NES cells were kept in proliferating conditions and passaged at least twice before the treatment with MeHg. After 48h of exposure, parent cells (P) were trypsinized to obtain daughter cells generation 1 (G1). G1 cells were differentiated and analyzed at differentiation day (DD) 21 or 28 (DD21 and DD28). Growth factors (GFs) were withdrawn 24 h after G1 plating. In proliferating G1 cells, intracellular ROS measurement, gene expression analysis, EdU assay and apoptosis index were determined 72 h after G1 plating. **b) Assessment of healthy NES susceptibility to MeHg at different low concentrations.** Apoptotic index in control and MeHg-treated cultures was evaluated in G1 cells after 3 days of proliferation using Hoechst 33342 to count nuclei with normal morphology and nuclei exhibiting apoptotic chromatin condensation. Significantly increased levels of apoptosis were found only in cultures exposed to 12.5 nM. Control cultures exhibited 3.65% apoptosis (n=3). **c) Effects of 10nM MeHg exposure on intracellular ROS level.** The ratio carboxy-DCFDA/Hoechst 33342, shown as relative fluorescence for control and MeHg-exposed cells, was determined in G1 cells after 3 days of proliferation (n=3). Error bars represent SEM, $*p \leq 0.05$.

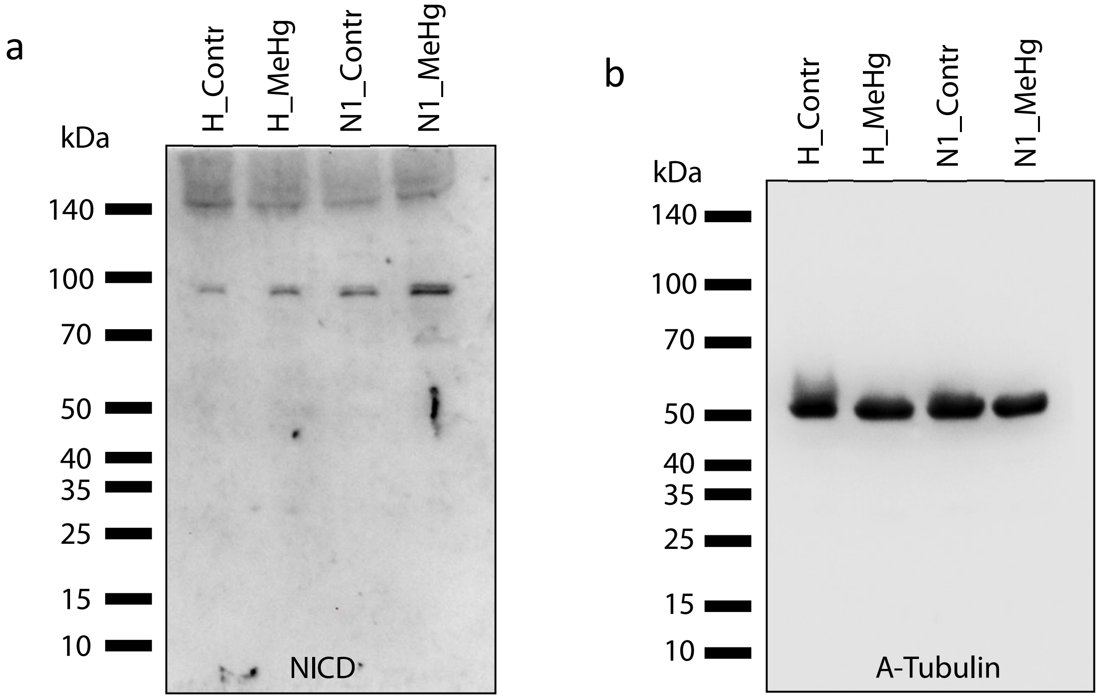

Supplementary Figure S2. Western blot evaluation of selected proteins. Full length blots for NICD (a) and alpha-Tubulin (b).
